# Intra-pericardial thrombin injection in iatrogenic cardiac tamponade: a case report

**DOI:** 10.1186/s43044-024-00459-5

**Published:** 2024-02-26

**Authors:** Juana Perez Morales, Ana Spaccavento, Santiago Ordoñez, Rocío Baro, Diego Conde, Juan Pablo Costabel

**Affiliations:** Department of Cardiology at Instituto Cardiovascular de Buenos Aires, Av. del Libertador 6302, 1428 Buenos Aires, Argentina

**Keywords:** Pericardial tamponade, Thrombin, Cardiac tamponade, Percutaneous intervention, Endomyocardial biopsy

## Abstract

**Background:**

Nowadays, percutaneous procedures are expanding in use, and this comes with complications associated with the procedure itself. Cardiac tamponade is rare but may be life threatening since it can involve hemodynamic instability. It is known that after pleural effusion during a percutaneous procedure, pericardiocentesis should be used as drainage of the cavity. However, that does not achieve hemostasis in some cases, and in those patients who are hemodynamically unstable, a sealing agent to promote hemostasis might be useful, like thrombin.

**Case presentation:**

We present a case report of 89-year-old patient with history of melanoma undergoing treatment with pembrolizumab, who attended the emergency department referring chest pain (intensity 5/10) and palpitations that have lasted hours. He had TnTUs 554/566 ng/L and an echocardiogram that showed dilated right chambers, hypertrophy and global hypokinesia of the left ventricle, increased filling pressures of the left ventricle and pulmonary hypertension. Myocarditis associated with pembrolizumab was suspected, so high dose steroids were initiated and endomyocardial biopsy was conducted, resulting in iatrogenic cardiac tamponade. To determine the etiology of the suspected myocarditis, an endomyocardial biopsy was performed. Unfortunately, an intraprocedural complication arose: pleural effusion resulting in iatrogenic cardiac tamponade, leading to hemodynamic instability. It required immediate pericardial drainage via subxiphoid puncture, obtaining a 550 mL hematic debit. Clinical manifestations raised suspicion of tamponade, prompting a bedside echocardiogram for a definitive diagnosis. Despite these efforts, the patient remained hemodynamically unstable, and due to the elevated surgical risk, intrapericardial thrombin was employed to achieve successful hemostasis.

**Conclusions:**

Cardiac tamponade is a life-threatening condition that can sometimes be induced iatrogenically, resulting from percutaneous interventions. Despite limited evidence regarding this therapeutic strategy, in patients experiencing iatrogenic cardiac tamponade with hemodynamic instability and high surgical risk, the administration of intra-pericardial thrombin could be contemplated.

## Background

The pericardium is a fibroelastic sac that surrounds the heart, and pericardial effusion occurs when the fluid accumulated is more than 50 mL. Cardiac tamponade happens when the pericardial fluid begins to accumulate, affecting the cardiac filling and reducing stroke volume, resulting in cardiogenic shock. Cardiac tamponade is a life-threatening condition, that is associated with different etiologies: idiopathic (presumed to be viral or immune-mediated), infectious (viral, bacterial), autoimmune and autoinflammatory, neoplasic, cardiac (myocarditis), trauma (including iatrogenic) and metabolic (uremia). Iatrogenic cardiac tamponade might be due to catheter and pacemaker perforations, cardiopulmonary resuscitation or as a complication of thoracic surgery. In a Swedish nationwide registry study, 44.497 underwent invasive electrophysiology procedures, and cardiac tamponade resulted in 200 patients [1].

## Case presentation

Male patient of 89 years-of-age, with history of smoking and arterial hypertension, and the following cardiovascular background: complete right branch bundle block, bilateral pulmonary embolism resulting in anticoagulation, atrial fibrillation, and heart failure with preserved ejection fraction. As mentioned before, he also had melanoma in current treatment with a checkpoint inhibitor (Pembrolizumab), and chronic renal disease (creatinine 2.2 mg/dL).

He was admitted in the emergency department because of chest pain of intensity 5/10 with palpitations and dyspnea. The physical examination revealed signs of left sided heart failure, and the patient exhibited no fever. Moreover, he remained lucid with no apparent neurological impairments. The following routine exams were carried out: Electrocardiogram: AF rhythm, 60 bpm, complete right bundle-brunch blockade; Chest X-ray cardiothoracic index increased/cardiomegaly, redistribution of pulmonary flow, and broad vascular pedicle. He had a high sensitive troponin T at admission of 554 ng/L and creatinine of 3 mg/dL. Echocardiogram showed right chambers dilated, left ventricle with global hypokinesia, increased left ventricle filling pressures and pulmonary hypertension. Ejection fraction was estimated in 40%.

**Fig. 1:**
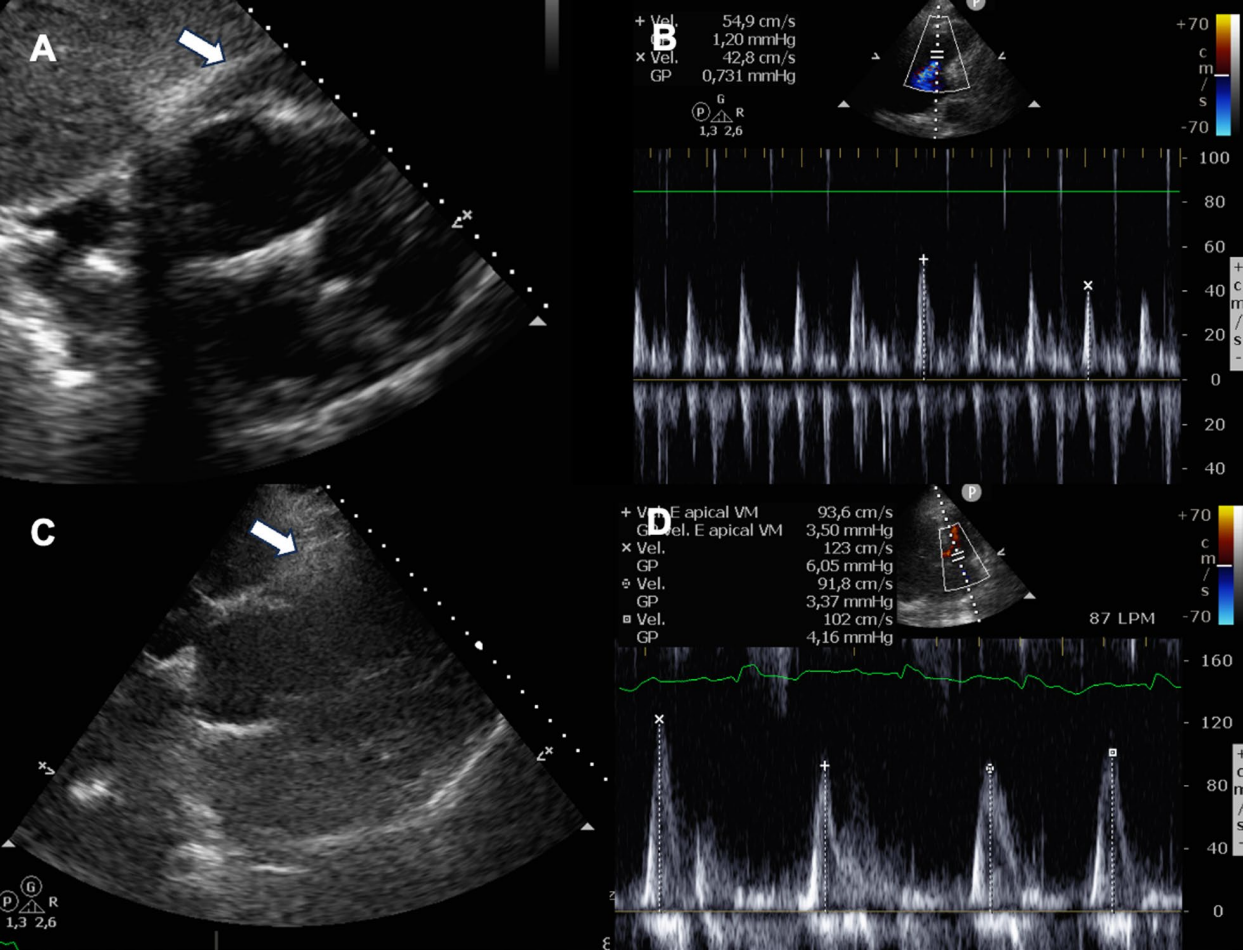
**A** 2D Echocardiogram: Subxiphoid view showing pericardial fluid with right ventricular collapse consistent with cardiac tamponade (arrow); **B** Doppler echocardiography: variation in mitral peak E velocity more than 25–40% with inspiration; **C** 2D Echocardiogram: Subxiphoid view showing no pericardial effusion (arrow); **D** Doppler echocardiography: mitral peak velocity without abnormal variation

Coronary artery disease was initially considered as a potential diagnosis; however, given the patient's medical history, and the temporal relationship with the administration of pembrolizumab, a drug related to the occurrence of myocarditis, that became the primary suspicion, leading to the initiation of high-dose steroid treatment. To determine the etiology of the suspected myocarditis, an endomyocardial biopsy was performed. Unfortunately, an intraprocedural complication arose: pleural effusion resulting in iatrogenic cardiac tamponade, leading to hemodynamic instability. The patient had a blood pressure (BP) of 70/40 mmHg and a heart rate (HR) of 130 bpm, with poor peripheral perfusion and mental confusion requiring volume and inotrope support with noradrenaline. Clinical manifestations raised suspicion of tamponade, prompting a bedside echocardiogram that confirmed diagnosis. It required immediate pericardial drainage via subxiphoid puncture, obtaining a 550 mL hematic debit. After pericardiocentesis, patient improved hemodynamically (BP 140/80 HR 100 bpm), but this did not last more than two or three minutes. The pericardial fluid was drained and re-generated rapidly. A catheter for pericardial drainage was placed and more than 1 L of blood drained in the first hour. He received three units of platelets as treatment since he had been on aspirin therapy for the past 48 h. Additionally, prothrombin complex 600 IU was administered due to the history of apixaban use more than 48 hs before. Aminocaproic acid (10 g in 100 mL) was administered, and four units of red blood cells were transfused. Despite these efforts, the patient remained hemodynamically unstable, and due to the elevated surgical risk, intrapericardial thrombin (600 UI of thrombin in 1 mL of saline) was employed, administered as a bolus, to achieve successful hemostasis. Echocardiograms (Fig. 1): the initial 2D echocardiogram before thrombin injection shows pericardial fluid and right ventricular collapse, indicative of cardiac tamponade. This goes along with the second image that shows the mitral peak E wave that has more than 25–40% of variation within inspiration, also consistent with cardiac tamponade. Contrarily, the resting images show no pericardial fluid accumulation after the intervention has been made, and the mitral peak E waves velocity within normal variation.

## Discussion

In this case report, we aim to discuss the use of intra-pericardial thrombin as a rescue strategy for achieving hemostasis in hemodynamically unstable patients experiencing iatrogenic cardiac tamponade. According to Holmes et al. [2], the incidence of cardiac tamponade in percutaneous cardiac interventions varies, occurring in up to 6% of procedures, and it is the most common potentially life-threatening complication of these kinds of procedures. Cardiac tamponade requiring pericardiocentesis is described in the following procedures: ICD (implantable cardioverter defibrillator), CRT (cardiac resynchronization therapy), pacemaker and PCI (percutaneous coronary intervention) [2–5]. It should be noted that cardiac tamponade associated with endomyocardial biopsy is not described in the literature. There is a case report published by Tong-Lee et. al, about thrombin use in left ventricle wall rupture following myocardial infarction, with favorable outcomes [6, 7].

In 2022, Rottländer et. al published a single-center retrospective cohort involving 31 patients, who presented cardiac tamponade after percutaneous intervention, and used intrapericardial thrombin injection as a bailout strategy in 5 of the 31 patients. Thrombin was administered when the other therapeutic options were exhausted (reversion of anticoagulation, medicinal circulatory support and treatment of the cause of the pleural effusion) and continued with hemodynamic instability. They used 5000 UI of thrombin in 250 mL of saline solution. Half of the patients had a percutaneous coronary intervention, 1/3 of the total of patients underwent cardiac implantable electronic device, but none had an endomyocardial biopsy. When comparing pericardial thrombin injection with standard treatment (surgery if needed), none of the 5 patients from the first group needed cardiothoracic surgery. During the 30-day follow-up, it was noted that the thrombin-injection group had prolonged intensive care unit stay and more severe bleeding, but mortality was lower in this group compared to standard treatment. None of the 5 patients underwent cardiac surgery, compared to 11.5% in the standard treatment group. Also, no complications were observed while using intra-pericardial thrombin, not even pericarditis symptoms [5].

## Conclusions

Cardiac tamponade is a life-threatening condition that can sometimes be induced iatrogenically, resulting from percutaneous interventions. Despite limited evidence regarding this therapeutic strategy, in patients experiencing iatrogenic cardiac tamponade with hemodynamic instability and high surgical risk, the administration of intra-pericardial thrombin could be contemplated.

## Data Availability

All data generated or analyzed during this study are included in this published article.

## Abbreviations

CRT: Cardiac resynchronization therapy
ICD: Implantable cardioverter defibrillator
PCI: Percutaneous coronary intervention
